# Underachievement Risks and Profiles of Psychological Variables Among High-Ability Adolescents from Hong Kong, The Netherlands, Taiwan, and The United Kingdom

**DOI:** 10.3390/ejihpe15090178

**Published:** 2025-09-04

**Authors:** Edmund T. T. Lo, Marjolijn van Weerdenburg, Joanne M. Williams, Enyi Jen, Lianne Hoogeveen, Stella W. Y. Chan, Kuen Fung Sin, Ho Nam Cheung

**Affiliations:** 1 Behavioural Science Institute, Radboud University, P.O. Box 9104, 6500 HE Nijmegen, The Netherlands; edmund.lo@ru.nl (E.T.T.L.); marjolijn.vanweerdenburg@ru.nl (M.v.W.); lianne.hoogeveen@ru.nl (L.H.); 2 Department of Clinical and Health Psychology, University of Edinburgh, Edinburgh EH8 9AG, UK; jo.williams@ed.ac.uk; 3 Bridges Graduate School of Cognitive Diversity in Education, Los Angeles, CA 91604, USA; enyi.jen@bgs.edu; 4 Charlie Waller Institute, School of Psychology and Clinical Language Sciences, University of Reading, Reading RG6 6BZ, UK; s.chan3@reading.ac.uk; 5 Department of Special Education and Counselling, The Education University of Hong Kong, Hong Kong SAR, China; kfsin@eduhk.hk; 6 Department of Social Work and Social Administration, University of Hong Kong, Hong Kong SAR, China

**Keywords:** adolescents, high ability, latent profile analysis, underachievement

## Abstract

Background: High-ability students, despite their potential, may underachieve academically. The existing literature suggests the presence of subtypes, such as perfectionistic or creative high-ability students, who underachieve for different reasons. However, empirical work identifying these profiles and linking them to underachievement remains limited. Methods: We analyzed self-reported data by 930 high-ability adolescents across Hong Kong, the Netherlands, Taiwan, and the United Kingdom. We conducted a pre-registered confirmatory latent profile analysis on five dispositions theoretically relevant to underachievement: creativity, academic self-efficacy, self-regulation, perfectionistic standards, and self-criticism. We examined how these profiles related to underachievement risk, measured by academic performance and self-perceived underachievement. Results: Four profiles emerged. Two aligned with underachievement-related theories, namely the “self-satisfied” profile (low self-criticism, high self-regulation and creativity; prevalent in Asia) and the “maladaptively perfectionistic” profile (high self-criticism but low creativity, academic self-efficacy, and self-regulation; prevalent in Western Europe). Academic performance did not differ across profiles. However, adolescents in the “self-satisfied” profile were less likely to self-perceive as underachievers, while those in the “maladaptively perfectionistic” group were more likely. Interestingly, self-perception as underachievers in both profiles was positively linked with academic performance. Conclusions: These findings provide empirical evidence on subtypes among high-ability students that may differentially present underachieving risks.

## 1. Introduction

High-ability students do not always perform academically up to their potential throughout their entire school career. This discrepancy between one’s high ability—often reflected in their receiving nominations to participate in enrichment programs—and actual performance in grades is often referred to as ‘underachievement’ (Jackson & Jung, 2022; Peterson & Jen, 2023; Steenbergen-Hu et al., 2020). Although selection methods to identify underachievement are diverse (Lau & Chan, 2001), Siegle (2018) estimated that up to 50% of all students who have high ability may underachieve at some point in their schooling. Underachievement in high-ability students is a loss for those students themselves and to society (Steenbergen-Hu et al., 2020). As a worldwide phenomenon seen across America, Europe, and Asia (Obergriesser & Stoeger, 2015; Dittrich, 2014), researchers and educators have been particularly keen to understand these students at an early stage in adolescence (Dai et al., 2011), before they demonstrate maladjustment in higher education (Honken & Ralston, 2013), risk dropping out of education (Mattern et al., 2015), and experience poor socioemotional development (Blaas, 2014; Castejón et al., 2016).

### 1.1. Underachieving High-Ability Adolescents: Different Subtypes, Reported, but Untested

There has long been a recognition that we need to move away from the assumption of “one size fits all” in education—especially in enrichment programs for high-ability students (Bondie et al., 2019). Research from clinical observations, interviews, and literature review has suggested numerous subtypes that characterize underachieving high-ability students (Mandel & Marcus, 1991; Delisle & Galbraith, 2002; Neihart & Betts, 2010; Ronksley-Pavia & Neumann, 2020; see review by Siegle, 2021). Two commonly discussed subtypes in the literature are: Students who disengage from academic activities because of the fear of failing to maintain their achievement (e.g., “Perfectionistic Pearl”, S. B. Rimm, 1997; S. Rimm, 2007), and students who appear creative and behaviourally non-confirming who do are not interested in the normal curriculum (“the Creative”, Neihart & Betts, 2010). These common subtypes, with good face validity in depicting underachieving high-ability students, have been taken into account in educational practice and policy for adolescents globally (ACT Education Directorate, Australia, 2021; CBO Nijmegen, 2024; Davidson Institute, 2020; Gifted Education Section, Education Bureau, the Hong Kong Special Administrative Region Government, 2020; Potential Plus UK, 2020).

Despite their influence in real-life practice, there has been scarce empirical support for the presence of these subtypes in adolescent populations. Within the limited research, there have been latent profile analyses that explored subtypes of U.S. students based on the Big Five personality traits (Cross et al., 2018; Mammadov et al., 2016; Mammadov, 2023). However, these findings are not easily generalized to high-ability adolescents outside the U.S. (Hernández-Torrano & Kuzhabekova, 2020). Moreover, the analyzed traits were not central to underachievement theories, offering limited insight into advancing our prior understanding of underachievement (Finch & Bronk, 2011). Thus, there is a need to perform theoretically relevant, confirmatory testing on subtypes of underachievement identified by profiling theoretically relevant psychological variables (Bauer, 2022) in adolescent students sampled from different regions. This approach enables researchers to interpret how these variables may interact and synergize, uncovering patterns that may contribute to underachievement.

### 1.2. Theories of Underachievement, Associated Psychological Variables, and Possible Profiles

Drawing on existing literature, we have identified three theoretical models that may be particularly informative in hypothesizing which psychological variables characterize high-ability adolescents and how these variables may relate to underachievement.

#### 1.2.1. Achievement Orientation Model

The Achievement Orientation Model (AOM; Siegle & McCoach, 2005) is the most prominent theoretical model of underachievement in high-ability students (Ritchotte et al., 2014; Steenbergen-Hu et al., 2020). It posits that students need to believe they have the ability to achieve (i.e., have academic self-efficacy; Bandura, 1977; Muris, 2001; Honicke & Broadbent, 2016) before they can prepare and sustain studying activities for attaining academic goals (i.e., self-regulation; Siegle & McCoach, 2005; Brigandi, 2015; Siegle et al., 2017; Moilanen, 2007; Zimmerman, 2013; McCoach & Siegle, 2003; Baslanti & McCoach, 2006). Consistent with this theoretical model, academic self-efficacy and self-regulation have been empirically shown to be positively related to academic performance among both general student populations (Artino, 2012; Multon et al., 1991; Boyd et al., 2005; Theobald, 2021) and high-ability students (Carr et al., 1991; Obergriesser & Stoeger, 2015), and self-regulation has been further suggested as an intervention target to improve academic performance (Labuhn et al., 2010).

Therefore, according to the AOM, a potential profile for high-achieving, high-ability adolescents would likely include high academic self-efficacy and self-regulation. This profile resembles “the autonomous learner” (Neihart & Betts, 2010).

#### 1.2.2. Maladaptive Competence Beliefs Pathway

The Maladaptive Competence Beliefs Pathway contends that perfectionism and underachievement go hand-in-hand (Cross, 1997; Glover et al., 2007; Snyder & Linnenbrink-Garcia, 2013; Mofield & Parker Peters, 2019): when students see that they cannot attain the perfect performance in their eyes, they avoid engaging in studying behavior. This disengagement serves to provide the individual with a behavioral excuse for poor performance that does not threaten their self-identity, which is deeply tied to performance.

Perfectionism comprises two factors, namely perfectionistic standards and self-criticism (Stoeber & Otto, 2006; Richardson & Rice, 2015). Perfectionistic standards refer to the performance expectations that one holds. Individuals who are high on self-critical perfectionism experience dissatisfaction and frustration upon failing to reach their standards and are at risk of reducing engagement and dropping out. Here, the self-criticism factor is central to testing the Maladaptive Competence Belief Pathway. However, the perfectionistic standards factor may also be relevant, as it has been traditionally theorized to facilitate higher academic achievement (Fong & Yuen, 2009; Madigan, 2019). For instance, one study showed that high-ability middle school underachievers show lower levels of perfectionistic standards compared with other high-ability students (Mofield et al., 2016). Given the complex relationship between perfectionism factors and academic achievement, featuring in both high achievement and underachievement (Madigan, 2019; Park et al., 2020), we decided to consider both perfectionistic standards and self-criticism in identifying subtypes of underachievers.

Thus, according to the Maladaptive Competence Beliefs Pathway, a possible profile of underachieving adolescents would be featured by high self-criticism; i.e., adolescents see themselves as unable to attain their academic goals, which indicates low academic self-efficacy. This profile resembles several reported subtypes of high-ability students, such as “Perfectionistic Pearl” (S. B. Rimm, 1997), “Stressed Learner” (Heacox, 1991), “Anxious Underachieving” (Mandel & Marcus, 1991), and the “successful learner” (Neihart & Betts, 2010).

#### 1.2.3. The Creative Students

Creative students can have high academic achievement (Gajda et al., 2017; Akpur, 2020), provided that their novel ideas are appropriate to classrooms and curricula (Miller, 2012; Sternberg, 2005). However, creativity can be a liability to student-teacher relationships. Students’ creativity might manifest itself in areas unexpected to teachers, for example, self-modifying assignments or engaging in their own activities (Kanevsky & Keighley, 2003; Thompson & McDonald, 2007; Kim, 2008; Kim & VanTassel-Baska, 2010; Figg et al., 2012). These activities could arise when students find conventional academic tasks unchallenging, which can happen frequently in high-ability students (Matthews & McBee, 2007; Lakin & Wai, 2020). If students’ creativity is seen as nonconformity in teachers’ eyes (Scott, 1999; Sztolpa et al., 2016), poor student-teacher relationships may follow. Such poor relationships are detrimental to students’ school adjustment and academic achievement (Freeman, 2016).

Therefore, one hypothesized profile of underachieving high-ability adolescents would be featured by high creativity. This possible profile resembles several reported subtypes of high-ability students, such as “Dominant Non-Conformer” (S. B. Rimm, 1997), “the Rebel” (Heacox, 1991), “Identity Search Underachiever” (Mandel & Marcus, 1991), and “the Creative Learner” (Neihart & Betts, 2010).

### 1.3. Underexamined Regional Differences

Regional differences in the education of high-ability students, sociodemographic variables, school systems, and cultural expectations regarding academic attainment are likely to play a role in influencing students’ psychological outcomes and academic performances. Indeed, cross-cultural differences in psychological variables have been reported in the general student population. Specifically, perfectionistic standards and self-criticism have been found to be more typically seen in Asian than Western students (W.-W. Chen et al., 2022; Kawamura, 1999). Self-regulation and creativity have been reported to be higher among Western students than in Asian students (R. T.-H. Chen, 2014; Dent & Koenka, 2016; G. Xie & Paik, 2019). These regional differences in psychological variables are likely to result in differences in the prevalence of profiles of variables among high-ability adolescents across regions; however, this hypothesis has yet to be examined.

### 1.4. The Present Study

To address the gaps identified in the field as discussed above, in the present study, we aimed to identify subtypes of high-ability adolescents by testing for profiles of psychological variables, namely academic self-efficacy, self-regulation, creativity, perfectionistic standards, and self-criticism. Specifically, we aimed to test three hypotheses:

Hypothesis 1 states that there will be more than one profile of psychological variables. Based on the three theoretical models discussed above, we further hypothesized that a confirmatory testing would result in three hypothesized profiles that featured some of the five psychological variables measured in our study: (a) A profile of high academic self-efficacy and self-regulation (H1A); (b) a profile of low academic self-efficacy and high self-criticism (H1B) and (c) a profile of high creativity (H1C).

Hypothesis 2 states that high-ability adolescents in different psychological profiles will differ in risks of underachievement, indicated by their academic performance (pre-registered analysis) and self-perception as underachievers (exploratory analysis). We then explored whether such self-perception was associated with academic performance similarly across profiles.

Hypothesis 3 entails three sub-hypotheses based on earlier reports of the cultural differences: There are more Asian adolescents in the H1A profile (H3A), and more Western adolescents in the H1B profile (H3B) and H1C profile (H3C). We chose to compare Asian and Western adolescents, rather than the four samples from Hong Kong, the Netherlands, Taiwan, and the United Kingdom, for two reasons. The first reason was to ensure sufficient sample sizes for latent profile analysis. The second reason was the limited research on differences between regions on the same continents. We present exploratory results on the nuanced regional differences.

## 2. Materials and Methods

The hypotheses and analysis plan of this study had been pre-registered at https://osf.io/6tcmk/?view_only=b064af9f2267456daf6d79aeaa56e4cd (accessed on 21 July 2025) before we analyzed the data (information on minor deviations is in Supplemental Materials S1). Our annotated codes and results have been deposited at the Open Science Framework (OSF)at https://osf.io/32dzu/?view_only=8654529c32a9464694eea0b9203028d5 (accessed on 21 July 2025) Data used in this study can be requested from the corresponding author.

### 2.1. Participants and Procedure

This study was a part of a larger international collaboration that studied high-ability adolescents in the Hong Kong Special Administrative Region, the Netherlands, Taiwan, and the United Kingdom, regions where the authors are based and have a deeper understanding of the respective education systems (project code: UGC/FDS16/H11/21). The procedures of this project were approved by the ethics board of Hong Kong Metropolitan University (HE-RGC2021/AS11). In 2022, students aged between 12 and 18 years from these four regions were recruited by Qualtrics via advertisements on school websites, social media, or through project members’ professional networks. Parental consent was sought prior to the administration of the questionnaires. A total of 3984 adolescents reported their demographic information, health information, history of being identified as high-ability students, history of participating in intelligence/cognitive ability tests, academic performance, and a battery of validated measures via an online questionnaire. Responding to each item was made compulsory, so there was no missing data. Participating adolescents received approximately 6 USD as compensation (exact amount unspecified, see Douglas et al., 2023).

This study only included adolescents who reported having been identified as having high ability, indicated by having answered ‘yes’ to at least one of these four questions: “Are you currently a member of any gifted/enrichment programme?”, “Have you been nominated for any gifted/enrichment programme in the past?”, “Are you receiving extra support from your school as the more-abled student?”, and “Did you receive extra support from your school as the more-abled student at any point in the past?” There were 1174 adolescents who answered “yes” to at least one of these four questions. To assess the appropriateness of using these screening questions, we inspected the proportion of these screened adolescents who have undergone intelligence or cognitive ability tests, which have been conventionally employed for identifying high-ability (Worrell & Erwin, 2011). Among the 590 adolescents (out of the N = 3984 full sample) who reported having ever taken those tests, 74% of them belonged to the 1174 screened adolescents, representing a disproportionately high percentage (*X*^2^(1) = 650.28, *p* < 0.001). Therefore, we considered it appropriate to use these four questions as a screening.

Among the 1174 screened adolescents, we only included 942 who belonged to the ethnic majority group. For example, those who self-identified as ‘Caucasians’ in Western European regions, but not those who self-identified as ‘Caucasians’ in Asian regions (see Clark et al., 2012). This strategy was deemed to be necessary to maximize the homogeneity among adolescents from the same regions for clearer regional comparisons. We excluded 12 adolescents who gave irrelevant responses in open-text questions regarding their academic performance (e.g., answering “who is it” in response to a question about their English subject performance). The final sample size therefore consisted of 930 12–18-year-old adolescents (age *M* = 16.27, *SD* = 1.63, female = 44%, male = 55%, non-binary = 1%; Table 1). We had sufficient power per continent to perform our planned analyses so that fit indices in latent profile analysis would function well (Nylund-Gibson & Choi, 2018).

### 2.2. Measures

All measures in English were translated and back-translated for each region when the validated translations were unavailable. The traditional Chinese versions (for Hong Kong and Taiwan adolescents) were translated by the bilingual research team. All translations were performed based on Brislin’s (1986) back translation model. Bilingual co-authors then checked both Chinese and Dutch versions.

#### 2.2.1. Underachievement Risks

##### Poor Academic Performance

Adolescents’ poor academic performance was measured by their percentage of poor-to-average school grades. The online questionnaire presented a list of subjects that adolescents studied (e.g., language subjects, science, and mathematics). Adolescents indicated yes/no to whether they performed poorly or not on each subject they studied based on criteria applicable to different regions, for example, “fail” (all regions), “score 60 or lower” (Hong Kong), and “grade 6 or lower” (The Netherlands). We calculated the percentage of poor performance out of all subjects for each adolescent for further analysis. The higher this percentage is in an adolescent, the greater their risk of underachievement (Niederdeppe, 2009).

##### Self-Perception as Underachievers

Adolescents answered a yes/no question on “Do you consider yourself as an underachiever?”. A description was provided to ensure clarity: “Underachievers can be interpreted as someone who does not show their learning potential in their school performance. In other words, their performance does not match their cognitive ability/intelligence.” This definition was adapted from previous literature (Snyder & Adelson, 2017; Lavrijsen et al., 2022).

#### 2.2.2. Psychological Variables for Latent Profile Analysis

For all variables, we reported the omega reliability with the observed sample variance based on the confirmatory factor analyses we have conducted. We interpreted reliability above 0.70 as sufficiently high (McNeish, 2018).

##### Creativity

We used the Creativity Subscale of the Renzulli Scales (Renzulli, 2021). This scale showed convergent validity with divergent thinking and artistic involvement (Chan & Zhao, 2010) and has been rated as higher among high-ability adolescents (Labăr & Frumos, 2013). A sample item was “Able to generate different ideas (e.g., able to think of different uses of an object).” The 11 items, rated on a 6-point Likert scale from “never see myself with this characteristic” (1) to “always see myself with this characteristic” (6), had sufficient reliability (Asian: 0.931, Western: 0.897).

##### Academic Self-Efficacy

We used the Academic Subscale of the Self-Efficacy Questionnaire for Children (SEQ-C; Muris, 2001). Previous validation studies confirmed the factor structure of SEQ-C, which supported our use of the Academic Subscale (H. Xie et al., 2022). Additionally, the Academic Subscale showed good convergent validity with school satisfaction and academic performance (Suldo & Shaffer, 2007). A sample item was “How well do you succeed in understanding all subjects in school?” The 8 items of the academic subscale of SEQ-C, rated on a 5-point Likert scale from “not at all” (1) to “very well” (5), had sufficient reliability (Asian: 0.850, Western: 0.855).

##### Self-Regulation

We used the Adolescent Self-Regulatory Inventory (ASRI; Moilanen, 2007), which has been used in various adolescent samples (e.g., De Vet et al., 2014; Yıldız Durak, 2020). A sample item was “I can stay focused on my work even when it’s dull.” The 36 ASRI items, rated on a 5-point Likert scale from “not at all true for me” (1) to “really true for me” (5), had sufficient reliability (Asian: 0.877, Western: 0.846).

##### Perfectionistic Standards

We used the Standard Subscale of the Almost Perfect Scale-Revised (APS; Slaney et al., 1996). The factor structure of the Almost Perfect Scale-Revised has been replicated across various samples, validating the reliability of its subscales (Vandiver & Worrell, 2002; Chan, 2011). Two sample items of the Standard Subscale were “I have high expectations for myself” and “I have high standards for my performance at work or at school.” The seven items, rated on a 7-point Likert scale from “strongly disagree” (1) to “strongly agree” (7), had sufficient reliability (Asian: 0.812, Western: 0.837).

##### Self-Criticism

We used the 12-item Discrepancy Subscale of the Almost Perfect Scale-Revised (APS; Slaney et al., 1996). Two sample items of the Discrepancy Subscale were “I rarely live up to my high standards.” and “I hardly ever feel that what I’ve done is good enough.” This subscale had sufficient reliability (Asian: 0.889, Western: 0.903).

#### 2.2.3. Data Analysis

All analyses in this study were conducted in the R computing statistical language (R Core Team, 2023).

##### Measurement Invariance

To ensure consistent measurement of constructs across regions, we performed multi-group confirmatory factor analysis (MGCFA) on a final sample of 930 participants. This allowed us to assess measurement invariance between Asian and Western European participants regarding the five psychological variables. For each scale, we tested three MGCFA models to examine different levels of invariance: configural (where items load in the same direction for both groups), metric (where items have consistent loadings between groups), and scalar (where group-level means are equivalent). According to recent practices, achieving metric and scalar invariance ensured meaningful comparisons across regions (Greco et al., 2022). After MGCFA, we summed all items in a variable to obtain its sum score.

##### Standardization Across Regions

We standardized the five psychological variables using the mean and *SD* of the full sample of 930 adolescents, allowing Z-scores to be easily compared across variables. Regional standardization was unnecessary, as MGCFA results confirmed measurement invariance across regions. However, poor academic performance was standardized as a Z-score, and self-perception as underachievers was mean-centered within each region to enable meaningful cross-regional comparisons.

##### Latent Profile Analysis (Testing Hypothesis 1)

We followed the latest recommendations in conducting latent profile analysis and used the functions from the tidySEM package (Van Lissa et al., 2023) for testing all hypotheses. Specifically, we used the mx_profiles() function to run latent profile analysis on mixture models from the five psychological variables we measured. For hypothesis 1, we anticipated that three profiles would emerge. Therefore, we set the maximum number of profiles in mx_profiles() to four. We used the default analysis settings of tidySEM and its dependency OpenMX (Neale et al., 2016): the SLSQP optimizer with a gradient step size of 0.00001 and the global optimizer simulated annealing, ensuring outcomes are independent of starting values. Full details of the analysis setup can be retrieved at https://osf.io/32dzu/?view_only=8654529c32a9464694eea0b9203028d5 (accessed on 21 July 2025).

Several iterations in latent profile analysis were run with an increasing number of profiles. Upon convergence, we inspected fit indices to determine whether the model with one, two, three, or four profiles best described the data. We primarily preferred the model with the lowest Bayesian Information Criterion (BIC), which balanced model fit and model complexity (Berlin et al., 2014). Furthermore, we inspected the entropy of each model, a probability of whether participants belong in one particular profile, hence indicating the separability of profiles. We considered 0.6 as a minimally required level (Weller et al., 2020). In addition, we followed previous recommendations to only accept models with sizes of profiles of at least 50 adolescents or 5% of the total sample (Weller et al., 2020). We considered 10 as the acceptable threshold for the global observation-to-parameter ratio and the local participant-to-parameter ratio in evaluating a model (Kline, 2023).

##### Auxiliary Analyses (Testing Hypotheses 2 and 3)

To test hypothesis 2 (where we compared the underachievement risks across identified profiles) and hypothesis 3 (where we compared the regional differences across different profiles), we used two methods: the likelihood ratio test and the BCH method (Bolck et al., 2004), which controlled for classification errors. To further reveal differences between specific profiles in a certain outcome variable, we used Wald tests, which were implemented via the wald_test() function. We adopt a conventional threshold of *p* < 0.05 for testing hypotheses 2 and 3.

## 3. Results

### 3.1. Descriptive Statistics

#### 3.1.1. Underachievement Risks

The regional average of poor academic performance, defined as the percentage of poor-to-average school grades across all subjects, was 47.7% in Western Europe (43.6% in the United Kingdom and 53.3% in the Netherlands) and 67.2% in Asia (65.6% in Hong Kong and 68.4% in Taiwan). Regarding self-perception as underachievers, 45.6% of adolescents in Western Europe identified as such (36.6% in the United Kingdom and 57.5% in the Netherlands), compared to 17.5% in Asia (18.3% in Hong Kong and 16.8% in Taiwan).

#### 3.1.2. Psychological Variables for Latent Profile Analysis

Descriptive statistics of psychological variables are shown in Table 2. The full breakdown by region is reported in Supplemental Materials S2. Descriptive data suggested that the data were kurtotic (peaked) and generally left-skewed (for visual inspection of probability distributions, see Supplemental Materials S2). These were attributable to some participants’ tendency to rate the minimum score for all items in a scale. However, there was no compelling evidence indicating that these participants provided invalid responses. They displayed variance in responses to some scales (e.g., consistently rating 1 on creativity but varying their responses on the ASRI). Moreover, they did not uniformly provide the lowest rating on the ASRI, which includes reverse-coded items. As a result, we did not exclude any participants on this basis.

### 3.2. Measurement Invariance

The changes in fit indices from MGCFA models that assumed configural, metric, and scalar invariance were sufficiently modest that we could assume scalar measurement invariance between Asian and Western European participants (Supplemental Materials S3). Therefore, we proceeded with the planned latent profile analysis.

### 3.3. Latent Profile Analysis Revealed Four Profiles Among High-Ability Adolescents

As pre-registered, we conducted latent profile analysis using four models that assumed one to four profiles. All models converged. Table 3 presents the fit indices for each model. None required disqualification according to thresholds of entropy, profile sizes, and observation/participant-parameter ratios.

**Figure 1:**
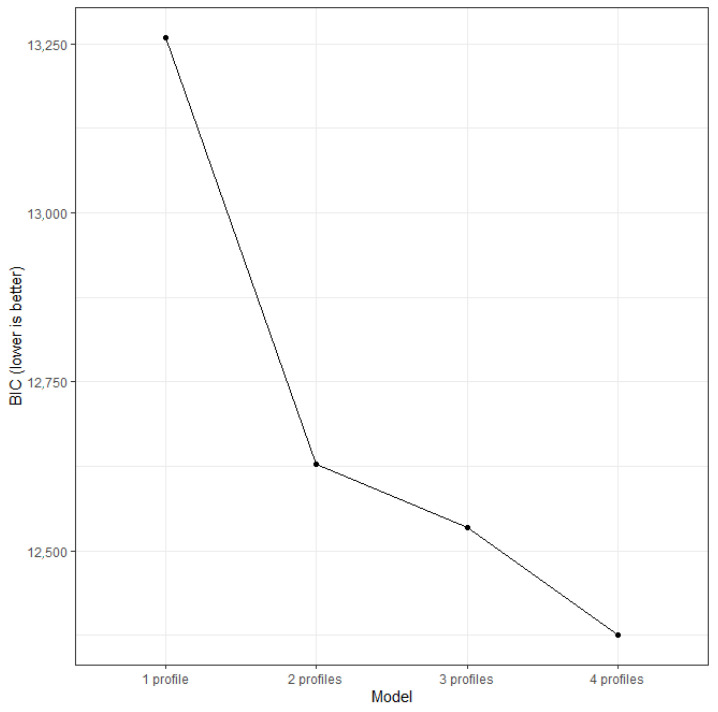
Scree plot of BIC of Models with one to four Profiles.

Likelihood ratio tests indicated that pairwise comparisons between models were significantly different. BIC is reduced with the increasing number of profiles. The four-profile model had the lowest BIC, indicating the best fit among models with one to four profiles. The four-profile model contributed a greater reduction in BIC than the three-profile model did, suggesting the four-profile model was preferred (Figure 1). In terms of interpretability, the four-profile model had an entropy of 0.78, which indicated the highest separability between profiles among models with more than one profile. The only drawback of the four-profile model was its smallest profile, comprising 8% (71 participants), smaller than the 10% in the three-profile model. However, this still exceeded the minimum criterion of 5% or 50 participants. We additionally inspected the distribution of psychological variables of profiles in the three-profile model against that in the four-profile model. The extra profile in the four-profile model met subhypothesis H1B, with the rest of the three profiles showing a similar distribution as the profiles in the three-profile model (Supplemental Materials S4). Therefore, we proceeded with the four-profile model solution because it presented the best fit, had the strongest interpretability, and offered an extra profile that matched theoretical expectations. Consistent with Hypothesis 1, our results suggested that high-ability adolescents did not represent a homogeneous group but instead were characterized by four subtypes.

### 3.4. Profiles Matched Expectations by Three Theoretical Perspectives

**Figure 2:**
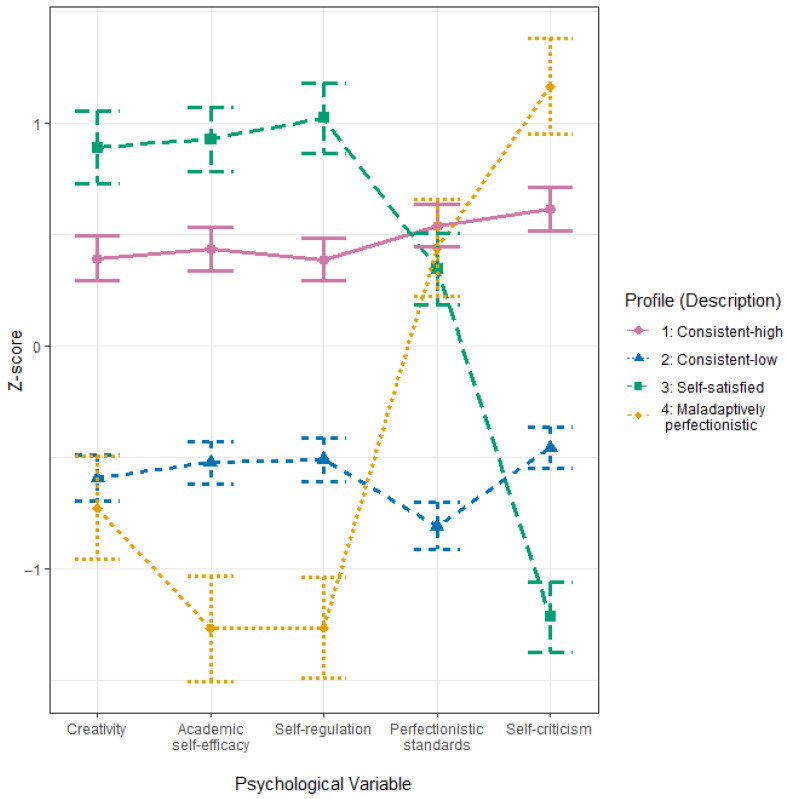
Profile Plots of Four Profiles Based on Psychological Variables Measuring Creativity, Academic Self-Efficacy, Self-Regulation, Perfectionistic Standards, and Self-Criticism.

Estimates of psychological variables in each of the four profiles are presented in Table 4 and Figure 2.

Profile 1, characterized by consistently high scores in all five psychological variables, can be described as *consistent-high*. Profile 2, characterized by consistently low scores in all five psychological variables, can be described as *consistent-low*. Profile 3, resonating with sub-hypotheses H1A and H1C, was indicated by the highest scores in creativity, academic self-efficacy, and self-regulation but the lowest score in self-criticism, and hence we hereby described this subgroup as *self-satisfied*. Profile 4, matching sub-hypothesis H1B, was featured by the highest score in self-criticism, the lowest academic self-efficacy, and the lowest self-regulation among all profiles, and may be described as *maladaptively perfectionistic*. It should be noted that, except for Profile 2, which reported the lowest score in perfectionistic standards among all profiles, perfectionistic standards did not significantly differ between the other three profiles.

### 3.5. Underachievement Risks (Self-Perceived Underachievement and Actual Academic Performance) Across Profiles

We examined adolescents’ underachievement risks with two measures: Their self-perception as underachievers (centered to regional mean) and actual poor academic performance as measured by percentage of poor-to-average grades (standardized by regional mean and *SD*). The BCH method (Bolck et al., 2004) indicated that the differences in self-perception as underachievers were significant among profiles, ∆LL(6) = 93.90, *p* < 0.001. Pairwise comparisons indicated that the ‘self-satisfied’ (Profile 3) adolescents were the least likely, and the ‘maladaptively perfectionistic’ (Profile 4) adolescents the most likely, to see themselves as underachievers (all *p*’s < 0.001 after false discovery rate correction for multiple comparisons; Benjamini et al., 2009). In contrast, there was no support in the presence of differences in percentage of poor-to-average grades between profiles, ∆LL(6) = 9.26, *p* = 0.159.

**Figure 3:**
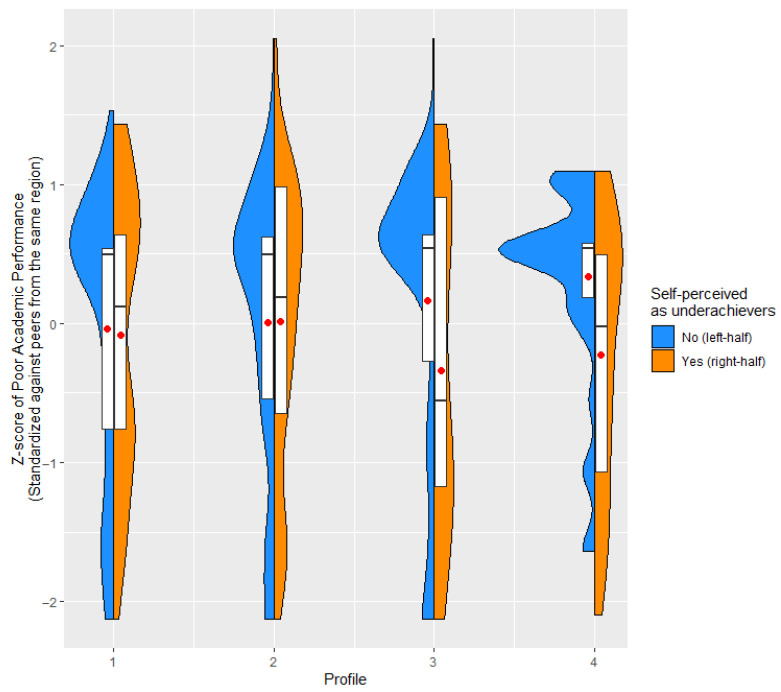
Violin Plots Showing the Distribution of Poor Academic Performance Across Four Profiles, Categorized by Adolescents’ Self-Perception as Underachievers. In each violin plot, the red dot represents the mean, the boxplot spans the first to third quartiles of the distribution, and the line within the box indicates the median.

Surprisingly, within these two profiles (“self-satisfied” and “maladaptively perfectionistic”), self-perception as underachievers acted as a buffer against poor grades for adolescents in two of the four profiles (see Figure 3). The association between perceiving oneself as an underachiever and actual academic performance differed significantly across profiles (∆LL(6) = 15.98, *p* = 0.001). Specifically, for adolescents in the ‘self-satisfied’ (Profile 3) and ‘maladaptively perfectionistic’ (Profile 4), the self-perception as underachievers was associated with a lower Z-score of poor academic performance, with regression coefficients of −0.80 and −0.69, respectively. These negative associations indicate that in Profiles 3 and 4, students who saw themselves as underachievers tended to perform better academically. All pairwise comparisons between Profiles 3 or 4 and Profiles 1 and 2 were statistically significant (all *p*’s < 0.05) after applying the false discovery rate correction for multiple comparisons (Benjamini et al., 2009).

### 3.6. Regional Differences in Profiles

Contrary to sub-hypothesis 3A, there were significantly more Asian participants among the ‘self-satisfied’ (i.e., Profile 3, characterized by the highest self-regulation) (70%) than in the overall sample (54%), *χ*^2^(1) = 17.41, *p* < 0.001. Contrary to sub-hypothesis 3B, there were significantly fewer Asian adolescents among the ‘maladaptively perfectionistic’ (i.e., Profile 4, characterized by the highest self-criticism) (29%) than in the overall sample (54%), *χ*^2^(1) = 24.08, *p* < 0.001. Sub-hypothesis 3C expected fewer Asian participants to be in profiles featured with the highest creativity. Profile 3 (‘self-satisfied’) had the highest creativity among all profiles. From sub-hypothesis 3A, we knew that there were more Asian participants in Profile 3 (‘self-satisfied’) than in the sample. Therefore, sub-hypothesis 3C was not supported.

Our exploratory analyses compared regions within the same continents (Supplemental Materials S5). In Asia, there were more Profile 1 (‘consistent-high’) adolescents from Taiwan than in Hong Kong (*χ*^2^(1) = 42.66, *p* < 0.001), and in Western Europe, more Profile 4 (‘maladaptively perfectionistic’) adolescents from the United Kingdom than from the Netherlands (*χ*^2^(1) = 48.41, *p* < 0.001).

## 4. Discussion

Using a diverse sample of 930 adolescents from Hong Kong, the Netherlands, Taiwan, and the United Kingdom, this study provides empirical evidence that high-ability adolescents are far from a homogeneous group. Instead, they cluster into four distinct psychological disposition profiles. These findings align with prior literature suggesting that there are subgroups of high-ability students, and they may be differentially at risk of underachievement. In particular, adolescents in the “self-satisfied” and “maladaptively perfectionistic” profiles were, respectively, less and more likely to perceive themselves as underachievers. Unexpectedly, those who self-identified as underachievers within these two profiles reported better actual academic performance.

### 4.1. Psychological Profiles of High-Ability Adolescents Identified

Two of the four profiles uncovered—labeled “self-satisfied” and “maladaptively perfectionistic”—might be especially of interest to researchers and educators. The “self-satisfied” profile is characterized by low self-criticism paired with high levels of all other measured attributes (creativity, academic self-efficacy, self-regulation, and perfectionistic standards). In other words, these adolescents set high standards and feel capable and creative, yet remain uncritical of themselves.

The “maladaptively perfectionistic” profile featured high perfectionistic standards and self-criticism coupled with low creativity, low academic self-efficacy, and poor self-regulation. These adolescents appear to strive for perfection while harshly criticizing themselves, feeling ineffective, and struggling to manage their learning. It is arguably a distressing combination of psychological dispositions.

These findings offer new insights into the heterogeneity of high-ability adolescents. Notably, some profiles exhibit characteristics that align with more than one theoretical framework. For example, the “self-satisfied” profile reflects both the Achievement Orientation Model, which highlights the importance of self-efficacy and self-regulation for academic success, and the creative-student perspective, which suggests that highly creative students might suffer from poor student–teacher relationships and suffer from lower motivation in academic tasks. Although these frameworks predict contrasting academic outcomes, adolescents in the “self-satisfied” profile did not systematically differ in academic performance from those in other profiles. Similar arguments can be made with other profiles, where levels of self-efficacy, self-regulation, and creativity were generally comparable.

Given that these three dispositions were positively correlated across the sample and were at similar levels within each of the four profiles, future theoretical work may consider how creativity can be explained or incorporated under the Achievement Orientation Model. A revised framework might also reconsider how these dispositions interact to influence academic performance, as informed by our current findings of the absence of differences in actual academic performances between profiles.

### 4.2. Underachievement Risks: Perceived Underachievement Versus Actual Academic Performance

At the profile level, there were differences in self-perceived underachievement. However, actual academic performance did not differ systematically across the four profiles. With the “self-satisfied” and “maladaptive perfectionistic” profiles, a different pattern emerged: adolescents who perceived themselves as underachieving had better academic performance compared to the rest of the adolescents in the same profile. Two interpretations of this association are possible, depending on whether there is actual underachievement.

If self-perception reflects actual underachievement, it may indicate that students with such self-perception possess exceptionally high potential, as they are underachieving despite performing above average compared to their peers. In this case, additional support could help them reach their full academic potential. If self-perception does not reflect actual underachievement, it may reflect unrealistic beliefs about achieving even higher performance than they already demonstrate. This interpretation appears to fit the ‘maladaptively perfectionistic’ (Profile 4) adolescents well. In this case, students might benefit from support in managing expectations and stress, setting realistic goals, and prioritizing growth over flawless performance.

To clarify these interpretations, future research should employ quantitative methods, such as analyzing past performance records and cognitive ability tests, to better assess students’ academic potential. Additionally, qualitative methods involving interviews with adolescents, their parents, and teachers could provide deeper insights into their experiences, functioning, and the psychological factors influencing underachievement.

### 4.3. Regional Differences in Profiles and Underachievement Risks

Our findings also reveal notable regional differences in how psychological profiles manifest, particularly in relation to students’ perceptions of underachievement. The “self-satisfied” profile was disproportionately composed of Asian adolescents, who made up approximately 70 percent of this group. Adolescents from this profile were the least likely to identify as underachievers. One possible explanation lies in cultural norms around self-presentation. In collectivist societies such as Hong Kong, Taiwan, or China, individuals often avoid statements or behaviors that could lead to “loss of face” (Keevak, 2022). As such, a high-ability student who is objectively performing well may refrain from labeling themselves as an underachiever, even if they privately acknowledge unrealized potential, in order to maintain an image of competence.

In contrast, the “maladaptively perfectionistic” profile, a profile in which high-ability adolescents were most likely to self-perceive as underachievers, was predominantly composed of Western European adolescents. This stands in contrast to earlier literature suggesting that perfectionistic dispositions are more prevalent in Asian cultures (W.-W. Chen et al., 2022; Kawamura, 1999). It is possible that among high-ability adolescents, a subgroup of the general adolescent population, these cultural patterns differ from general trends.

In addition to cultural differences between Asian and Western adolescents, future research on regional differences in these profiles may want to consider the systematic differences in education systems. One of such systematic differences is high-ability student identification. For example, in Taiwan, national science fair performance is crucial (Jen & Moon, 2015), while the Dutch government recommends schools to attend to the needs of high-ability students without giving specific identification guidelines (The Government of the Netherlands, 2020). The differences between regions in the same continent, such as the much lower prevalence of ‘maladaptively perfectionistic’ (Profile 4) adolescents in the Netherlands compared to the United Kingdom, may also point to avenues of future research to understand the influences of more nuanced cultural and systematic differences in high-ability student identification and education.

### 4.4. Implications to Schools

In light of our results, there are two practical implications. First, enrichment programs may benefit from adjustments tailored to the psychological dispositions presented by each subgroup (i.e., personalized intervention, García-Martínez et al., 2021). Our study provides a solid empirical basis for the implementation. For example, students in the “maladaptively perfectionistic” profile (Profile 4) may benefit from greater emphasis on setting realistic goals and managing self-criticism. To identify which of the four profiles students are most likely to belong to, teachers and school psychologists can conduct pre-assessments before offering enrichment interventions. Specifically, they may use our results and the publicly available code at https://osf.io/32dzu/?view_only=8654529c32a9464694eea0b9203028d5 (accessed on 21 July 2025) to assist in estimating students’ profile likelihoods.

The second implication concerns psychoeducation. Building on the first implication, psychoeducation can be provided to enhance teachers, parents, and students’ understanding of high-ability students. Our study (a) highlights that high-ability students consist of subgroups with different combinations of psychological dispositions, (b) suggests that strong academic performance does not necessarily indicate the absence of underachievement (notably in the “self-satisfied” and “maladaptively perfectionistic” profiles), and (c) implies that high-ability students may differ in psychosocial needs, given their different profiles of psychological dispositions. Raising awareness of these differences is important, as such needs could be easily overlooked by teachers or parents who assume that academic success reflects overall adjustment to schooling (Pfeiffer & Stocking, 2000).

### 4.5. Limitations

Several limitations of the present study can be mentioned. Firstly, as a cross-sectional study, this research cannot confirm the developmental pathways leading to underachievement or establish any causal relationships. The stability and malleability of these profiles are also uncertain (e.g., Racz et al., 2016; Kassis et al., 2022). Secondly, our inclusion criterion of students who attended enrichment programs might exclude high-ability adolescents who have consistently underachieved and were not invited to such programs. This restricts the generalizability of our findings. Future studies could verify our results by incorporating other nomination methods like peer or self-nomination (Jackson & Jung, 2022). Thirdly, instead of using objective data like school records, we relied on adolescents’ self-reported grades, which may have introduced larger measurement error. This was a necessary trade-off in using an online questionnaire for data collection and reaching the planned sample size across four regions. Fourthly, adolescents self-reported psychological variables and self-perception as underachievers, but teachers and parents are also important information sources (Siegle et al., 2020; Lavrijsen et al., 2022). Fifth, while we included psychological dispositions theoretically relevant to underachievement, we did not capture all variables emphasized in the referenced models (e.g., goal valuation in the AOM). Future studies could replicate our findings on subtypes by adopting a robust theory-driven data collection design and exploring gender differences. Sixthly, our analysis included only ethnic majority participants, potentially limiting insights into regional differences that might emerge from including ethnic minorities. Cultural differences in high-ability adolescents’ experiences in education may vary across demographics and environments within regions. Finally, this study focused on adolescents aged 12 to 18, yet underachievement is also possible in higher education (Schuur et al., 2020). Further studies could investigate whether similar profiles emerge in later education stages.

## 5. Conclusions

Using a cross-cultural sample of 930 high-ability adolescents, we showed that there were four subtypes of high-ability students featured by four profiles with different combinations of psychological dispositions. The ‘self-satisfied’ and ‘maladaptively perfectionistic’ profiles might be of particular interest for further research, as adolescents in these profiles had different self-perceptions as underachievers than other profiles. Furthermore, adolescents in these two profiles who self-perceived themselves as underachievers paradoxically had better academic performance. Although the needs of each of the four profiles described in this study await further research, our study contributes to the understanding of high-ability adolescents. They can belong to distinct profiles of psychological dispositions and differentially present underachievement risks.

## Figures and Tables

**Table 1:** The final sample of adolescents (N = 930).

	N	Age *M* (*SD*)	Female	Male	Non-Binary
Asian	498	16.10 (1.73)	37%	63%	0%
Hong Kong	219	15.93 (1.84)	40%	60%	0%
Taiwan	279	16.24 (1.63)	34%	66%	0%
Western European	432	16.46 (1.49)	52%	45%	2%
The Netherlands	186	16.52 (1.50)	37%	63%	1%
The United Kingdom	246	16.38 (1.47)	64%	32%	4%

**Table 2:** Descriptive Statistics and Correlations of Five Centered Psychological Variables (as Participants’ Self-perceptions) Used in Latent Profile Analysis (N = 930).

						Correlations			
Psychological Variable	Median	Min	Max	Skewness	Kurtosis	1	2	3	4
1. Creativity	0.07	−3.17	1.93	−0.46	3.14				
2. Academic self-efficacy	0.01	−3.24	1.97	−0.27	2.89	**0.522**			
3. Self-regulation	0.03	−3.48	2.49	−0.31	3.36	**0.476**	**0.543**		
4. Perfectionistic standards	−0.02	−3.74	1.91	−0.30	2.85	**0.360**	**0.318**	**0.300**	
5. Self-criticism	0.02	−2.99	2.22	−0.12	2.65	0.011	−*0.100*	−*0.092*	**0.407**

Note. All standardized scales had *M* = 0 and *SD* = 1. Italic coefficients indicate correlations significant at *p* < 0.05. Bolded coefficients indicate correlations significant at *p* < 0.001.

**Table 3:** Fit Indices of Latent Profile Analysis Results.

Model (Number of Profiles)	Log-Likelihood	n	Parameters	BIC	Entropy	prob min	n min	np ratio	np local
1	−6595.56	930	10	13,259.48	1.00	1.00	1.00	93.00	93.00
2	−6259.12	930	16	12,627.61	0.70	0.90	0.46	58.13	50.00
3	−6192.13	930	22	12,534.63	0.73	0.66	0.10	42.27	13.65
4	−6092.07	930	28	12,375.52	0.78	0.79	0.08	33.21	12.16

Note. BIC = Bayesian information criteria; n = number of observations (participants); prob min = probability of the most likely profile membership by true profile membership, which indicates minimum classification reliability; n min = minimum percentage of participants assigned to each profile; np ratio = global ratio of observations per parameter; np local = observations per parameter in the smallest class.

**Table 4:** Four-Profile Model: Percentage of Profile Membership, Estimates of Psychological Variables, Percentage of Asian, Percentage of Female, Estimates of Age, and Poor Academic Performance.

Variable	All Participant	Profile 1 (Consistent-High)	Profile 2 (Consistent Low)	Profile 3 (Self-Satisfied)	Profile 4 (Maladaptively Perfectionistic)
Profile Membership	100%	40%	38%	14%	8%
(% in the overall sample)					
Psychological Variables ^a^					
Creativity	0.00 (1.00)	0.39 (0.62)	−0.59 (0.62)	0.89 (0.62)	−0.73 (0.62)
Academic Self-Efficacy	0.00 (1.00)	0.43 (0.66)	−0.53 (0.66)	0.93 (0.66)	−1.27 (0.66)
Self-regulation	0.00 (1.00)	0.38 (0.62)	−0.51 (0.62)	1.02 (0.62)	−1.27 (0.62)
Perfectionistic standards	0.00 (1.00)	0.54 (0.73)	−0.81 (0.73)	0.34 (0.73)	0.44 (0.73)
Self-criticism	0.00 (1.00)	0.61 (0.78)	−0.46 (0.78)	−1.22 (0.78)	1.16 (0.78)
Poor Academic Performance ^b^	0.00 (1.00)	−0.06 (1.01)	0.01 (0.98)	0.11 (1.04)	0.05 (0.85)
Self-perception as underachievers (% in the profile, not mean-centered) ^c^	31%	31%	31%	11%	57%
Asian (% in the profile) ^c^	54%	49%	58%	70%	29%
Female (% in the profile) ^c^	45%	50%	38%	38%	61%
Age ^d^	16.27 (1.63)	16.45 (1.47)	16.06 (1.76)	16.20 (1.71)	16.44 (1.49)

Note: ^a^ Means (*SD*s) in Z-scores standardized across all participants. ^b^ Means (*SD*s) in Z-scores standardized per region, i.e., benchmarked against peers from the same region. ^c^ No *SD* is shown as the percentage indicates the proportion of the variable. ^d^ Means (*SD*s), controlled for classification error.

## Data Availability

The raw data that support the findings of this study are available from the corresponding author upon reasonable request. This study’s analyzed data, code, and output are available at https://osf.io/32dzu/?view_only=8654529c32a9464694eea0b9203028d5 (accessed on 21 July 2025). The authors also provide an online page that details steps for predicting the likelihood of which profile a high-ability adolescent belongs to, at https://github.com/taktsun/HighAbilityProfile (accessed on 21 July 2025).

## Supplementary Materials

The following supporting information can be downloaded at: https://www.mdpi.com/article/10.3390/ejihpe15090178/s1, Figure S1: *Probability Distribution of 930 Participants’ Mean-centered Scores in Five Psychological Variables*; Table S1: Descriptive Statistics of Five Indicator Variables (as Participants’ Self-perceptions) in Latent Profile Analysis, Broken Down by Regions (N = 930); Table S2: Fit indices of multi-group confirmatory factory analysis on five indicator variables; Table S3a: Model results, percentage of poor-to-average grades, and percentage of region of three profiles; Table S3b: Number of Participants in Each Profile in the Three-Profile Model (Profile I to III) and Four-Profile Model (Profile 1 to 4); Table S4: Regional Prevalence of Each Profile Controlling for Classification Error.

## Abbreviations

The following abbreviations are used in this manuscript:

AOM	Achievement Orientation Model
APS	Almost Perfect Scale
ASRI	Adolescent Self-Regulatory Inventory
BIC	Bayesian Information Criterion
MGCFA	multi-group confirmatory factor analysis
SEQ-C	Self-Efficacy Questionnaire for Children
