# Supplementary material for: Underachievement Risks and Profiles of Psychological Variables Among High-Ability Adolescents from Hong Kong, The Netherlands, Taiwan, and The United Kingdom
Source: Eur J Investig Health Psychol Educ. 2025 Sep 4;15(9):178. doi: 10.3390/ejihpe15090178 (PMC12468528; doi:10.3390/ejihpe15090178)
Supplement: Supplementary file 1 [file ejihpe-15-00178-s001.zip › ejihpe-3801684-supplementary.pdf]

Underachievement Risks and Profiles of Psychological Variables Among High-Ability Adolescents from Hong Kong, The Netherlands, Taiwan, and The United Kingdom

Supplemental Materials

Edmund T. T. Lo, Marjolijn van Weerdenburg, Joanne, M. Williams, Enyi Jen, Lianne Hoogeveen,

Stella W. Y. Chan, K. F. Sin, and H. N. Cheung

Supplemental Materials S1: Deviations from Pre-registration

In our pre-registration, Hypothesis 2 stated the profile characterized by high self-regulation and academic self-efficacy had lower risk than the profile characterized by high creativity. This can only be tested if the profile featured by high creativity is not at the same time featured by high self-regulation and academic self-efficacy. However, results from latent profile analysis showed that participants in Profile 3 reported higher academic self-efficacy, self-regulation, and creativity compared to the other three profiles (as shown by the non-overlapping 95% confidence intervals of these variables in Profile 3; Figure 1). Therefore, this pre-registered hypothesis could not be tested. In our article, we tested a weaker form of the original hypothesis, namely adolescents from different profiles of psychological variables do not have the same academic performance. Furthermore, to deepen our understanding of underachievement risks among high-ability adolescents, we conducted exploratory analyses examining self-perception as underachievers and its association with adolescents' actual academic performance across profiles.

Supplemental Materials S2: Descriptive Statistics by Regions

Figure S1

Probability Distribution of 930 Participants' Mean-centered Scores in Five Psychological Variables

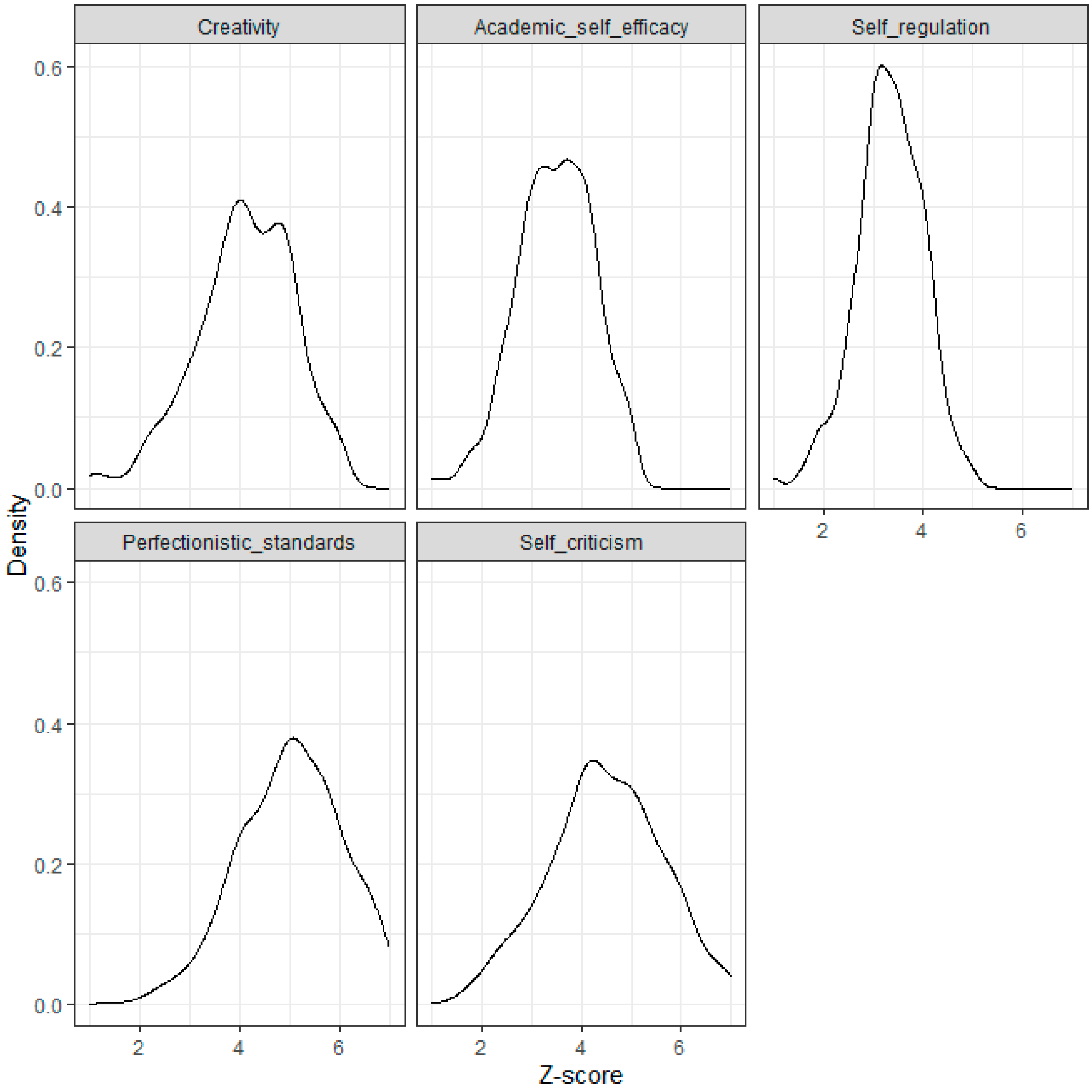

Table S1

Descriptive Statistics of Five Indicator Variables (as Participants' Self-perceptions) in Latent Profile Analysis, Broken Down by Regions (N = 930)

Scale	Hong Kong	Taiwan	The Netherlands	The United Kingdom	Total
			M (*SD*)		
Creativity	3.94 (0.99)	4.19 (1.05)	4.20 (0.88)	4.11 (0.95)	4.11 (0.98)
Academic self-efficacy	3.47 (0.74)	3.66 (0.71)	3.63 (0.75)	3.21 (0.79)	3.49 (0.77)
Self-regulation	3.43 (0.67)	3.44 (0.66)	3.29 (0.66)	3.15 (0.65)	3.33 (0.67)
Perfectionistic standards	4.8 (0.99)	4.93 (0.94)	4.94 (1.03)	5.37 (1.11)	5.02 (1.04)
Self-criticism	4.11 (1.11)	4.38 (1.04)	4.57 (1.11)	4.84 (1.17)	4.48 (1.13)

Supplemental Materials S3: Measurement Invariance

Table S2

Fit indices of multi-group confirmatory factory analysis on five indicator variables

	Chi-square	Degrees of freedom	CFI	TLI	RMSEA [90% confidence interval]	SRMR
Creativity						
Configural invariance	43.478	88	1.000	1.005	0.000 [0.000, 0.000]	0.027
Metric invariance	58.097	98	1.000	1.004	0.000 [0.000, 0.000]	0.031
Scalar invariance	73.173	108	1.000	1.003	0.000 [0.000, 0.000]	0.035
Academic self-efficacy (a subscale under the three-factor model of Self-Efficacy Questionnaire for Children)						
Configural invariance	307.833	454	1.000	1.005	0.000 [0.000, 0.000]	0.036
Metric invariance	434.427	474	1.000	1.001	0.000 [0.000, 0.007]	0.043
Scalar invariance	482.618	494	1.000	1.000	0.000 [0, 0.014]	0.045
Self-regulation						
Configural invariance	235.821	238	1	1	0.000 [0, 0.018]	0.043
Metric invariance	317.115	254	0.994	0.994	0.023 [0.014, 0.031]	0.05
Scalar invariance	336.768	270	0.994	0.994	0.023 [0.014, 0.031]	0.051
Perfectionistic standard and self criticism (two subscales under the three-factor model of Almost Perfect Scale-Revised)						
Configural invariance	1191.085	454	0.960	0.955	0.059 [0.055, 0.063]	0.072
Metric invariance	1367.616	474	0.951	0.948	0.064 [0.06, 0.068]	0.077
Scalar invariance	1413.57	494	0.950	0.949	0.063 [0.059, 0.067]	0.079

Note. Perfectionistic standards and self-criticism are two subscales in Almost Perfect Scale-Revised (APS). We ran a 3-factor model for APS. CFI = comparative fit index; TLI = Tucker Lewis index; RMSEA = root mean square error of approximation; SRMR = standardized root mean square residual.

Supplemental Material S4: Results of Three-Profile Solution

Hypothesis 1 expected three profiles, but not four which was suggested by the fit indices of latent profile analysis. Interpretability of the three-profile model and the four-profile model were compared (Table 4 and Table S3a). Overall, the profiles in the 3-profile model showed similar patterns as three of the four profiles in the 4-profile model. Similarity of profiles between the two models is also reflected in the cross-tabulation of the profile membership of participants in the three-profile model against that in four-profile model (Table S3b). The majority of the profile membership was a one-to-one mapping from 3- to 4-profile model, except for the first profile in the 3-profile model where a quarter of its members were redistributed to a new profile in the 4-profile model. A more detailed inspection on the entropy, estimates and profile plots revealed that the profiles in the three-profile solution had more overlapping profile-means in indicator variables. In other words, the profiles in the three-profile model are less distinctive between each other than those in four-profile model. In view of the fit indices and interpretability of profiles, we continued the rest of the hypotheses testing, results, and discussion based on the four-profile solution.

Table S3a

Model results, percentage of poor-to-average grades, and percentage of region of three profiles

	Profile I	Profile II	Profile III	All participants
	Estimated mean (*SD*) in z-score			
Model results				
Creativity	0.56 (0.79)	-0.73 (0.79)	-0.52 (0.79)	
Academic Self-Efficacy	0.58 (0.77)	-0.64 (0.77)	-0.81 (0.77)	
Self Regulation	0.48 (0.85)	-0.59 (0.85)	-0.53 (0.85)	0.00 (1.00)
Perfectionistic Standards	0.41 (0.77)	-0.92 (0.77)	0.55 (0.77)	
Self-criticism	0.04 (0.91)	-0.43 (0.91)	0.90 (0.91)	

Table S3b

Number of Participants in Each Profile in the Three-Profile Model (Profile I to III) and Four-Profile Model (Profile 1 to 4)

Profile	I	II	III
1	342	10	18
2	31	316	7
3	128	0	0
4	0	10	66

Supplemental Material S5: Regional Prevalence of Each Profile Controlling for Classification Error

Table S4

Regional Prevalence of Each Profile Controlling for Classification Error

Region	All participants	Profile 1 (consistent-high)	Profile 2 (consistent-low)	Profile 3 (self-satisfied)	Profile 4 (maladaptively perfectionistic)
Asian	53.55%	48.81%	58.00%	70.09%	28.78%
Hong Kong	23.55%	14.05%	28.50%	40.58%	17.73%
Taiwan	30.00%	34.76%	29.50%	29.51%	11.05%
Westen European	46.45%	51.19%	42.00%	29.91%	71.22%
Netherlands	20.00%	24.45%	19.84%	15.12%	8.28%
United Kingdom	26.45%	26.74%	22.16%	14.79%	62.94%

Note. The regional prevalences across the four profiles are exploratory findings. Differences in regional prevalence across four regions and profiles were found to be significant, $\Delta LL(12) = 142.11$, $p < .001$. Pairwise comparisons between profiles, adjusted for multiple comparisons using false discovery rate correction, revealed distinct regional prevalences for each profile. ($p < .05$ for all comparisons; Benjamini et al., 2009).
